# Evaluation of a novel technology for newborn resuscitation: A visual display of time since birth, video–audio recording, and ergonomic resuscitation equipment: A prospective observational design

**DOI:** 10.1177/20552076261431443

**Published:** 2026-04-02

**Authors:** Omkar Basnet, So Yeon Joyce Kong, Helge Myklebust, Sunil Mani Pokharel, Ashish KC

**Affiliations:** 1 Research Division, Golden Community, Lalitpur, Nepal; 2 54018 Strategic Research, Laerdal Medical, Stavanger, Norway; 3 59 Department of Obstetrics and Gynaecology, Bharatpur Hospital, Chitwan, Nepal; 4 70712 School of Public Health and Community Medicine, Institute of Medicine, University of Gothenburg, Gothenburg, Sweden; 5 Department of Women's and Children's Health, Uppsala University, Uppsala, Sweden

**Keywords:** Machine Learning Application (MALA), real-time feedback, ventilation, quality improvement

## Abstract

**Objective:**

Despite advancements in technologies, the quality of intrapartum care has consistently not improved. This study evaluates the potential efficacy of a novel technology for newborn resuscitation, which provides a visual display of time since birth, video–audio recording, and ergonomic resuscitation equipment, on healthcare providers’ performance during ventilation in Nepal.

**Method:**

This study utilized a prospective observational design conducted over 3 years at a referral hospital in Nepal. All infants who did not cry within 30 seconds of birth were included, and their ventilation performance was assessed across two phases: SUSTAIN (baseline phase) and Pre-MALA (pilot implementation phase). Ventilation performance was measured through direct observation and video annotation, with the median time to first ventilation compared between the two phases using the Mann–Whitney U test and generalized linear mixed model regression.

**Results:**

A total of 164 newborn ventilation events were observed, with 78 during the SUSTAIN phase and 86 during Pre-MALA phase. Direct observation was done in both phases, while video-recording annotation was also conducted during Pre-MALA phase. The median time to first ventilation significantly decreased from 84.3 seconds (interquartile range (IQR): 55.4–114.0) during SUSTAIN to 48.2 seconds (IQR: 33.5–85.0) during Pre-MALA (p < 0.001). The duration of suctioning before ventilation was reduced by 17.8 seconds (adjusted coefficient = −17.8; 95% CI; −23.1, −11.8) and time to first ventilation was reduced by 33 seconds (adjusted coefficient = −33.2; 95% CI; −51.1, −15.4) during Pre-MALA.

**Conclusion:**

The result suggests that novel technology during resuscitation can reduce time to first ventilation and unnecessary suctioning in a clinical setting. Further large-scale evaluations are needed to fully assess the potential impact on neonatal care.

## Background

In the past 20 years, despite a reduction in global neonatal mortality burden from 4.0 million in 2004 to 2.3 million in 2023, the burden of intrapartum-related neonatal mortality has not substantially decreased.^1,2^ In 2023, intrapartum-related mortality burden accounted for over 40% of global neonatal deaths, largely due to inadequate neonatal resuscitation care.^3^ Every year, an estimated 3.0 million neonates worldwide (3% of total births) require ventilation at birth,^4,5^ yet only 1 in 10 of these high-risk neonates receive timely and effective ventilation.^6^ It is crucial to improve healthcare providers’ (HCP) ability to provide high-quality ventilation care to neonates who are not breathing after the first steps of resuscitation in order to reduce neonatal mortality.^7^

Despite the availability of periodic updates to standard protocols for neonatal resuscitation, there has been significant deviation in care, especially in resource-constrained settings.^8,9^ To improve HCPs’ performance, various quality improvement (QI) strategies, such as medical education, simulation-based training,^10^ outreach-based sessions,^11^ reminders, and audits and feedback,^12^ have been recommended by the Cochrane Effective Practice and Organization of Care (EPOC) group.^13^ Several implementation studies have shown that outreach-based sessions, reminders, and audit feedback can improve HCPs’ clinical performance, but sustaining project-based QI strategies has been a challenge.^14–16^

In 2023, Nepal witnessed the effects of the inverse care law,^17^ where an unprecedented increase in institutional births combined with limited HCPs led to intrapartum mortality related to poor care.^18^ Over the past two decades, institutional childbirth in Nepal has increased from 8% in 2001 to 78% in 2023, without a corresponding increase in HCPs to manage the additional institutional births.^19,20^ As a result, neonatal mortality stagnated at 21 per 1000 live births between 2018 and 2023. Different implementation studies have shown that resuscitation technologies combined with audit and feedback can improve HCPs’ performance.^21^

Based on evidence that reminders and feedback during and after resuscitation can improve HCPs’ performance, we are conducting an innovative project called the Machine Learning Application (MALA). The MALA project aims to develop technological solutions that provide real-time guidance to HCPs during newborn resuscitation through automatic video and audio analysis and activity recognition using a deep learning model.^22^ A pilot version of the technology has been developed, which comprises a tablet mounted onto an infant warmer for video and audio recording, along with a visual display of the time since birth on the tablet monitor. The infant warmer is also equipped with resuscitation equipment, including suction, newborn heart rate monitor, and upright bag, providing ergonomic advantages by ensuring resuscitation tools are readily accessible for HCPs. This study evaluates the potential efficacy of this novel technology for newborn resuscitation with a visual display of time since birth on HCPs’ performance during ventilation in Nepal.

## Method

### Design

A prospective observational study design was used to assess the changes in HCPs’ ventilation performance from before (Scaling Up Safer Birth Bundle Through Quality Improvement in Nepal (SUSTAIN) study) and after (Pre-MALA study) the implementation of pilot technology. Baseline data from the SUSTAIN study, collected at Bharatpur Hospital, were compared with performance metrics from the Pre-MALA study. The baseline observation was done between 4 November 2019 to 20 June 2021 during the SUSTAIN. The SUSTAIN project was a comprehensive QI initiative to empower HCPs to enhance intrapartum and immediate postpartum care.^23^ The second observation (pilot implementation phase) was done between 24 November 2021 to 22 February 2023 during the Pre-MALA study. The Pre-MALA had two phases: Pre-MALA Phase I (from 24 November 2021 to 13 February 2022) and Pre-MALA Phase II (From 26 July 2022 to 22 February 2023). There was no difference between Pre-MALA-1 and Pre-MALA-2 in terms of technology introduction. The reason for differentiating two subphases was Pre-MALA-1 received more support to introduce the technology and ensure that it functions well and Pre-MALA-2 was the implementation of the technology in the routine system.^24^ For our main analysis, we combined data from both phases of Pre-MALA, referring to them collectively as Pre-MALA.

### Project setting

The study was conducted in a public referral hospital, Bharatpur, in Kathmandu, Nepal. The hospital has an annual delivery rate of 12,610 and an estimated 900 to 1,200 neonates require resuscitation.^25^ For childbirth, the hospital has a delivery room for vaginal births and an operation theater for cesarean births. All 15-support staff and 30 HCPs working in the delivery unit were invited to participate in the research project. The support staff were trained to sterilize equipment (i.e. NeoBeat) and support HCPs during delivery and document resuscitate events. The HCPs have all been trained and certified in the Helping Babies Breathe program. Before and during Pre-MALA, there was no change in human resource and clinical protocol.

### Participant selection criteria

Infants who were delivered in the labor room were eligible to the study. Those who did not cry within 30 seconds after birth were enrolled in the study. Infants whose mothers agreed to be part of the study on using the video recording for resuscitation were included in the study and thus the technology was used among the consented mothers. Further, after the video recording of the resuscitation events, mothers were approached again for consent to use the videos for research purposes.

### Intervention package

**Figure 1.**
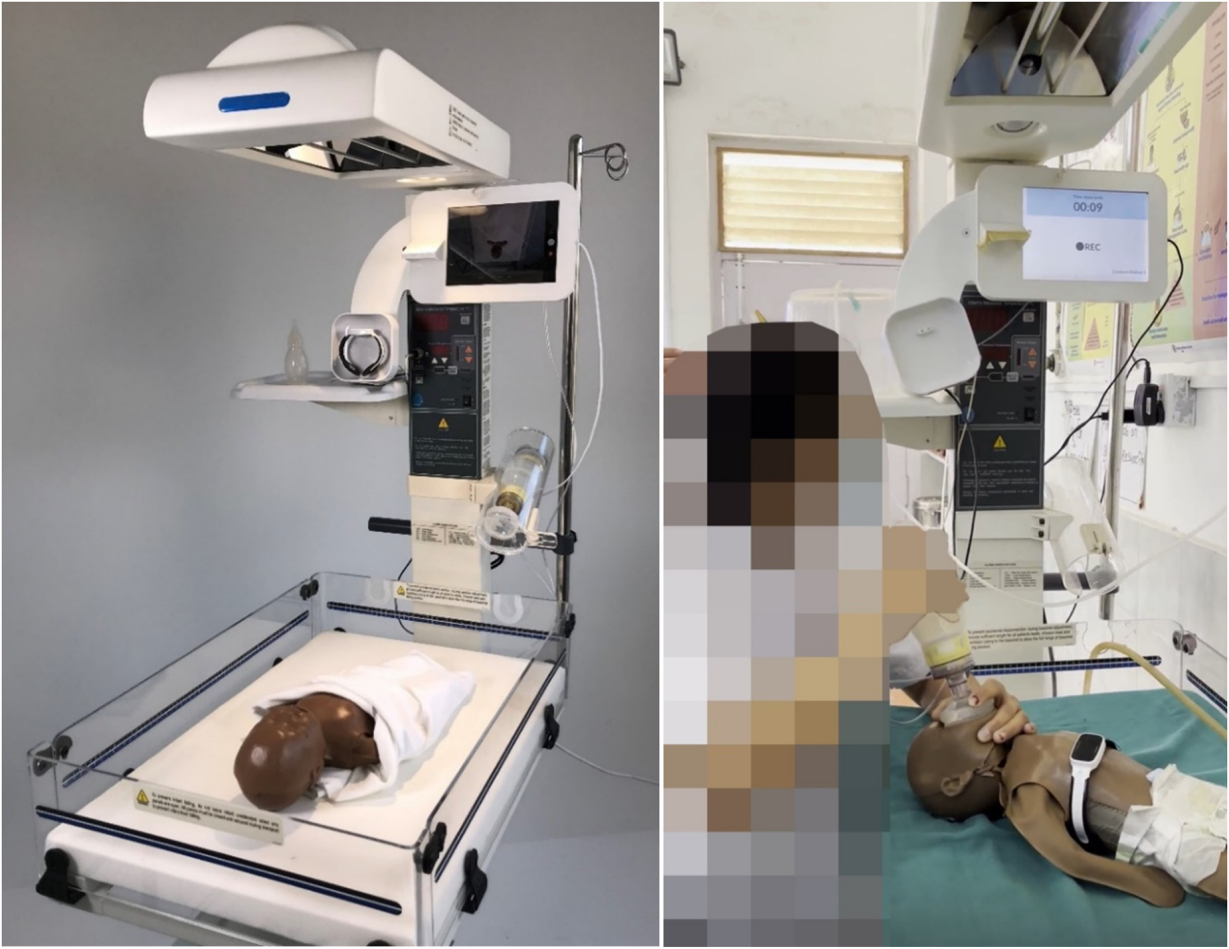
A pilot intervention technology used during Pre-MALA, featuring an infant warmer equipped with a mounted tablet computer, which includes a camera for video and audio recording, a NeoBeat, a manual suction device, and Upright bag with PEEP (left). An HCP in Bharatpur uses the technology to resuscitate a newborn manikin, monitoring the time since birth displayed on the tablet while being video and audio recorded (right). HCP: healthcare provider; MALA: Machine Learning Application; PEEP: positive end-expiratory pressure.

The pilot technology provided by Laerdal Global Health used during Pre-MALA consists of an infant warmer (Phoenix Medical Systems, Chennai, India) which is equipped with a tablet, that records video and audio of resuscitation activities and provides visual feedback on resuscitation in the elapsed time since birth. The infant warmer is also equipped with a Neobeat, a manual suction device, a neonatal resuscitator named Upright with PEEP (positive end-expiratory pressure). Neobeat is a dry electrode kept in placed on the baby's torso, which uses electrocardiogram (ECG)-based signal to measure heart rate. Upright bag and mask with PEEP is a self-inflating manual resuscitator that is intended for ventilatory support using reusable PEEP value to prevent alveolar collapse (Figure 1 *left*).

**Figure 2.**
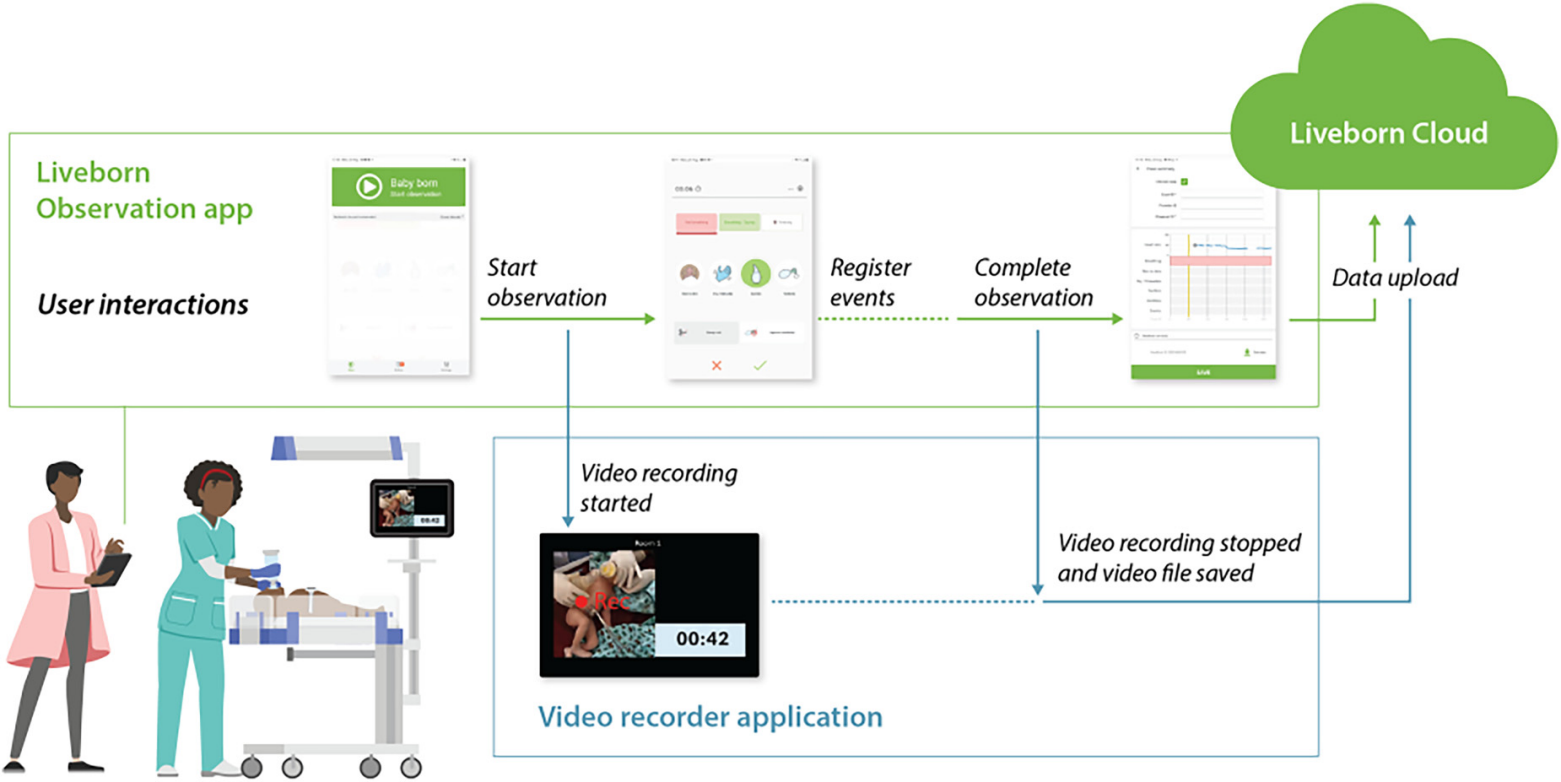
Overview of Liveborn applications, user interaction, and data flow during the Pre-MALA study. The time of birth and resuscitation activities are manually documented using the Liveborn Observation app. MALA: Machine Learning Application.

Newborn status and treatment were manually annotated using the Liveborn Observation application. Liveborn Observation application is a mobile application, used for research on newborn resuscitation, where an observer/data collector can document the timing of birth and resuscitation activities such as stimulation, skin-to-skin, clamp cord, suction, and ventilation by using push buttons.^26^ When the “baby born” button is clicked on the Liveborn Observation app at the time of birth, video recording is automatically started by the tablet mounted onto the infant warmer and elapsed time since birth is displayed on the tablet monitor (Figure 1). Newborn resuscitation events were observed and annotated in the Liveborn Observation app, and the newborn heart rate (HR) was streamed from Neobeat to the Liveborn Observation app. When no further resuscitative care was provided by HCPs, observation was ended in the Liveborn Observation app, and the video recording was also stopped automatically. Then both annotations and recorded video were uploaded to a highly secured Liveborn Cloud data storage system, hosted within Microsoft Azure. If a newborn did not need any resuscitative care after birth, the already initiated observation was canceled in Liveborn Observation app and the video recording stopped automatically and deleted from the data system (Figure 2).

### Data collection

For both study phases, the data collection was done through the Liveborn Observation app which records observed time of birth, resuscitation events, and outcome by trained independent research team (n = 4). Research team opened the Liveborn Observation app before birth and recorded the time of birth when birth was observed. Following birth, the support staff documented the start and stop times of key resuscitation actions including drying/stimulation, suction, and ventilation as well as the time of cord clamping using the Liveborn application. The support staff documented when the baby first cries and/or when the baby begins breathing well. If the newborn does not cry at birth, the HCPs place NeoBeat on the baby. NeoBeat synced with the Liveborn Observation app via Bluetooth technology as additional data input on the baby's condition. Data from the Liveborn Observation app and video recordings were stored and extracted from the Liveborn Cloud data storage system for further analysis.

The research team collected additional data on maternal demographics (maternal age and parity), intrapartum-related information (gestational age and intrapartum complications) and infants’ information (mode of delivery, birth weight, and outcome) from the hospital register.

### Data analysis

Descriptive statistics are presented as counts with percentages for categorical variables and median with range for continuous variables. p-Values were calculated using Pearson's Chi-square test for categorical and Mann–Whitney U test for continuous variables to compare the two study phases. We conducted generalized linear mixed model regression to assess the change in the performance between the two periods. To test temporal and interobserver variability in the way delivery and resuscitation activities were observed and annotated in the Liveborn Observation app by research team, we conducted sensitivity analysis by comparing time to first ventilation annotated in the Liveborn Observation app to ELAN (EUDICO Linguistic Annotator) video annotation tool.^27^ This tool played a vital role in managing the data collected during the study, ensuring precise annotation of video recordings and facilitating subsequent analysis. ELAN video annotation was conducted by a trained researcher according to a specific protocol, which was developed to ensure that each resuscitation activity or event was annotated according to the agreed-upon criteria set by our network of partner experts. The protocol specifies exact naming conventions for activities and events to guarantee accurate annotation for future artificial intelligence (AI) development. Data analysis was performed using SPSS Software (IBM Statistics 29) and STATA 16. All study-related procedures were conducted by the ethical standards enshrined in the Declaration of Helsinki.

## Results

**Figure 3.**
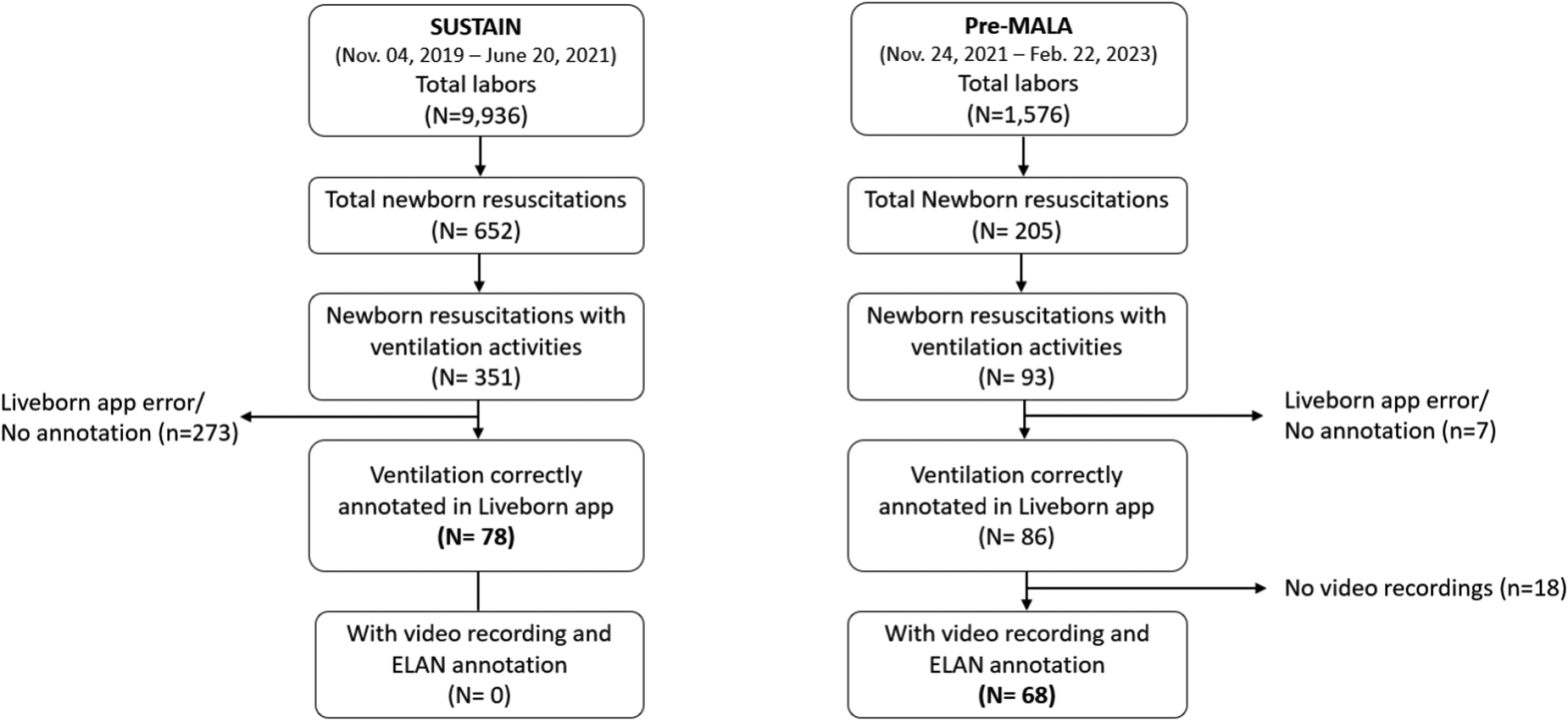
Flow diagram of included cases in the two study periods.

During each study phase, 351 (3.5%) and 93 (5.5%) newborn resuscitations with ventilation activities were observed for SUSTAIN and Pre-MALA, respectively. Of them, a total of 78 cases from SUSTAIN and 68 cases from Pre-MALA were included in the final analysis, after excluding cases without Liveborn Observation application annotation (n = 273 for SUSTAIN; n = 7 for Pre-MALA) and those without video recording and ELAN annotation among Pre-MALA cases (n = 18) (Figure 3). There was no difference in the background characteristics between the excluded and included population in each study phase (Supplemental Tables 1 and 2).

Median maternal age, gestational age, and birth weight were comparable between two study phases (p > 0.05 for all). Higher proportion of cases were nullipara among SUSTAIN cases (61.5%) compared with Pre-MALA (50.0%) cases (p = 0.004). There were statistically significant differences in maternal intrapartum complications (p < 0.001) and modes of delivery (p = 0.022) between two studies. Pre-MALA cases showed the highest proportions of intrapartum complications (88.2%) and assisted birth as mode of delivery (19.1%) compared with SUSTAIN (71.8% for intrapartum complications and 6.4% assisted births). No statistically significant difference (p = 0.793) was observed in newborn mortality between SUSTAIN (n = 1; 1.3%) and Pre-MALA (n = 1; 1.5%) study cases (Table 1).

**Table 1.** Demographic and clinical characteristics of study participants during SUSTAIN and Pre-MALA study periods.

Variables	SUSTAIN	Pre-MALA	p-Value*
	(N = 78)	(N = 68)	
Maternal age, median (IQR), years	25 (20–27)	51 (22–29)	0.435
Parity (%)			0.004
Nullipara	48 (61.5%)	34 (50.0%)	
Primipara	16 (20.5%)	23 (33.8%)	
Multipara	14 (17.9%)	11 (16.2%)	
Gestational age, median, (IQR), weeks	39 (38–40)	78 (37–40)	0.123
Intrapartum complications	56 (71.8%)	60 (88.2%)	<0.001
Mode of delivery (%)			0.022
Normal vaginal birth	73 (93.6%)	55 (80.9%)	
Assisted birth	5 (6.4%)	13 (19.1%)	
Birth weight, median (IQR), grams	3105 (2800–3407)	3076 (2655–3560)	0.816
Newborn mortality (%)			0.793
Live birth	77 (98.7%)	67 (98.5%)	
Death	1 (1.3%)	1 (1.5%)	

*p-Values are based on Mann–Whitney U test for continuous and Pearson's Chi-square test for categorical variables. IQR: interquartile range; MALA: Machine Learning Application; SUSTAIN: Scaling Up Safer Birth Bundle Through Quality Improvement in Nepal.

**Figure 4.**
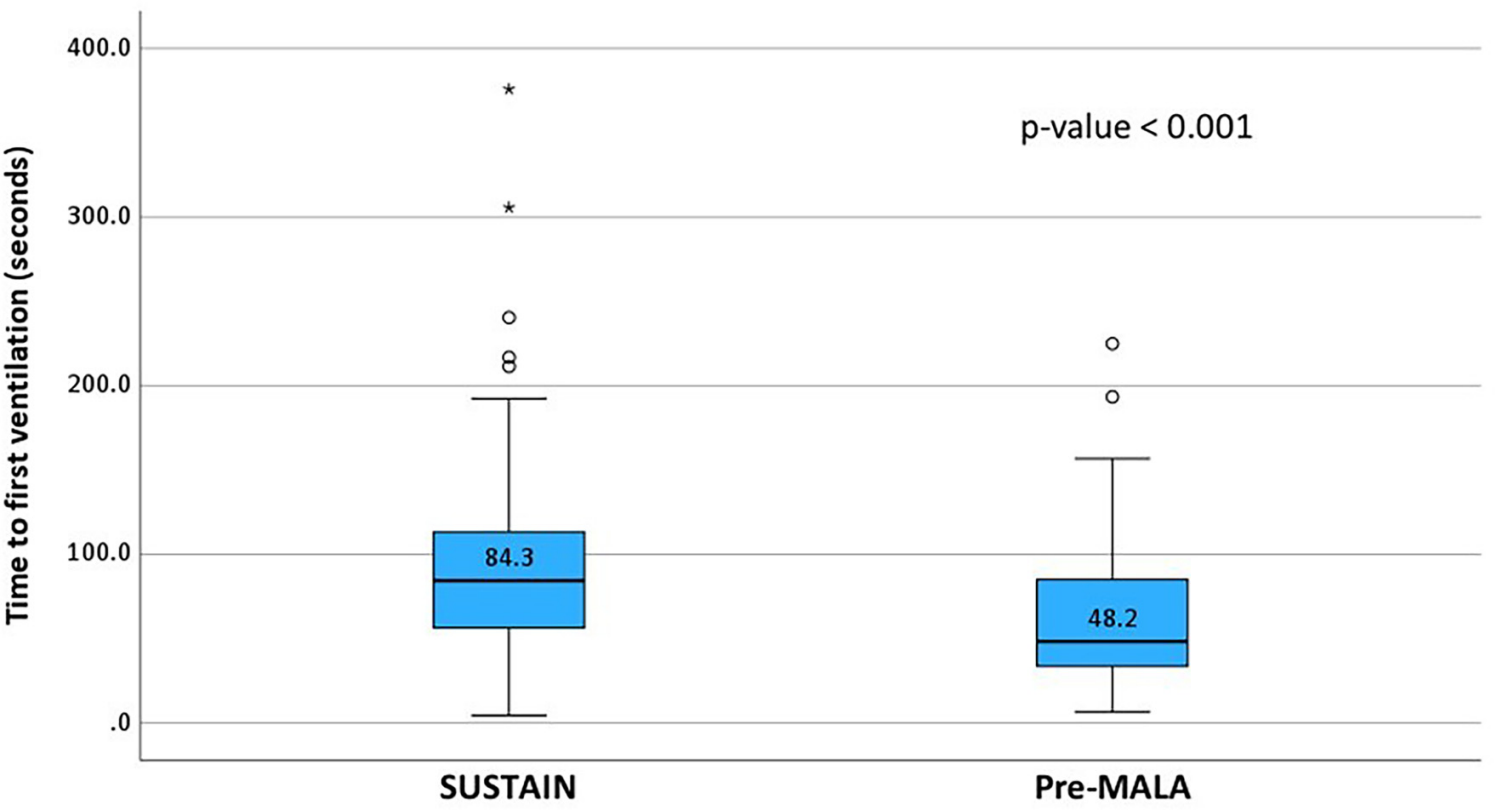
Boxplots with a median time (interquartile range) from birth to first ventilation (in seconds) between SUSTAIN (n = 78) and Pre-MALA (n = 68) groups based on the annotated time from the Liveborn Observation app. p-Value is based on the Mann–Whitney U test. MALA: Machine Learning Application

The median times to first ventilation were 84.3 seconds (IQR: 55.4–114.0) for SUSTAIN and 48.2 seconds (IQR: 33.5–85.0) for Pre-MALA cases with statistically significant longer median time to first ventilation in SUSTAIN cases compared with Pre-MALA cases (p < 0.001) (Figure 4).

The use and duration of suctioning before the first ventilation for participants is described in Table 2. The proportion of suctioning before the first ventilation was significantly higher within the SUSTAIN (75.6%) group compared to the Pre-MALA (41.2%) group (p < 0.001). The median duration of suctioning was 23.1 seconds (IQR 0.8–37.3) for SUSTAIN cases and 0 seconds (IQR 0–13.1) for Pre-MALA cases (p < 0.001). The mean suctioning duration was 24.4 seconds (SD 23.0) for SUSTAIN cases versus 6.6 seconds (SD 9.1) for Pre-MALA cases (p < 0.001) (Table 2).

**Table 2.** Use and duration of suctioning before first ventilation.

	SUSTAIN (78)	Pre-MALA (68)	p-Value*
Use of suction before first ventilation, n (%)	59 (75.6)	28 (41.2)	<0.001
Duration of suction before first ventilation in seconds			
Median (IQR)	23.1 (0.8–37.3)	0 (0–13.1)	<0.001
Mean (SD)	24.4 (23.0)	6.6 (9.1)	<0.001

*p-Values based on Chi-square for proportion, Mann–Whitney U test for median and t-test for mean. IQR: interquartile range; MALA: Machine Learning Application; n: number; SD: standard deviation; SUSTAIN: Scaling Up Safer Birth Bundle Through Quality Improvement in Nepal.

Duration of suctioning before ventilation was reduced by 17.8 seconds during the intervention period in comparison to the baseline period after adjusting with parity and intrapartum complication (adjusted correlation coefficient = −17.8; 95% CI; −23.1, −11.8). The duration of time to first ventilation was reduced by 33.2 seconds during the intervention in comparison to the baseline after adjusting with parity and intrapartum complication (adjusted correlation coefficient = −33.2; 95% CI; −51.1, −15.4) (Table 3).

**Table 3.** Generalized linear mixed model and regression model to assess the change in the duration and risk between the periods.

	Correlation coefficient change between the periods (95% CI)	
	Unadjusted	Adjusted^a^
Suctioning before ventilation (seconds)	−17.4 (−23.2, −11.6*, p* *<* *0.001*	−17.8 (−23.1, −11.8)*, p* *<* *0.001*
Time to first ventilation (seconds)	*−33.1 (−50.3, −15.8), p* *<* *0.001*	*−33.2 (−51.1, −15.4), p* *<* *0.001*

a Adjusted for parity and obstetric complication.

In sensitivity analysis, we compared the time difference between the first ventilation time based on Liveborn annotation and ELAN (video) annotation between two Pre-MALA phases. For Pre-MALA Phase I cases, the median time difference between Liveborn and ELAN annotations was 2.7 and 0.6 seconds for Pre-MALA Phase II cases with statistically no significant differences (p = 0.132), suggesting that there have been no significant temporal changes in the quality of Liveborn annotation (Table 4).

**Table 4.** Sensitivity analysis of time difference between first ventilation based on Liveborn annotation and ELAN (video) annotation between Pre-MALA Phase I and Pre-MALA Phase II.

	Pre-MALA Phase I (N = 20)	Pre-MALA Phase II (N = 48)	p-Value
Liveborn annotation, second, median (IQR)	38.6 (29.2–52.8)	51.5 (40.9–87.5)	
ELAN annotation, median (IQR)	37.5^a^ (28.9–28.5)	51.1 (36.6–86.6)	
Time difference between Liveborn and ELAN, median (IQR)	2.7^b^ (0.7–5.3)	0.6^c^ (0.2–1.5)	0.132

a For Pre-MALA Phase I study, ELAN annotations were performed by two researchers, therefore average of two ELAN annotation values was used.

b Based on absolute difference.

ELAN: EUDICO Linguistic Annotation; IQR: interquartile range; MALA: Machine Learning Application.

## Discussion

In this pilot study, we observed that innovative technology—integrating an infant warmer with a time display since birth for HCPs, along with the strategic arrangement of resuscitation equipment for easy access, and video/audio recording during newborn ventilation—had a positive effect on the workflow for managing nonbreathing newborns and can reduce the time to first ventilation. Before implementing this technology, the time to first ventilation was 84 seconds. After the introduction, this time was reduced by two-thirds to 38.6 seconds and remained less than 51 seconds even 1 year after implementation. Additionally, the use of suctioning before ventilation decreased from 75.6% during the SUSTAIN period to 41.2% after the implementation during Pre-MALA period. This significant reduction in unnecessary suctioning between two points suggests that displaying the time since the birth of the neonate on the monitor may have prompted a more rapid initiation of bag and mask ventilation.

Excessive or prolonged suctioning has consistently been identified as a significant factor contributing to delayed initiation of ventilation, particularly in low-resource settings. A study in Democratic Republic of Congo highlighted how such practice can lead to increased risks of adverse neonatal outcomes, including hypoxia and other complications, due to the critical delay in establishing effective ventilation.^28^ As observed in our study, novel technology may have helped reduce unnecessary suctioning, which likely contributed to the observed decrease in time to first ventilation, potentially mitigating some of the risks associated with delayed ventilation initiation.

Several factors may have contributed to the observed reduction in time to first ventilation: firstly, the real-time display of elapsed time since birth on the monitor likely served as crucial feedback for HCPs, encouraging them to initiate ventilation more promptly. This visual indication may have improved time awareness, and stimulated a sense of urgency, leading to quicker action during resuscitation. Secondly, the strategic placement of NeoBeat, the upright bag-mask ventilation (BMV) device, and suction equipment on the infant warmer helped streamline the resuscitation process, minimizing delays in accessing necessary equipment and thereby reducing the time to start ventilation. In the Pre-MALA survey study on usability, feasibility, and acceptability of the technology, 75% of the HCPs agreed that “the NeoBeat and upright bag & mask placed onto the infant warmer reduced the time to start the resuscitation.”^29^ In addition, the awareness of being recorded may have triggered the Hawthorne effect, where HCPs alter their behavior because they know they are being observed. While this study did not utilize video recordings for educational or quality improvement purposes, previous studies have shown that video recordings can enhance the quality of care through video reflection, debriefing, and feedback mechanisms.^30–33^

There was no difference in terms of the clinical protocol between SUSTAIN and Pre-MALA besides the technological intervention for display of time since birth and neonatal resuscitation duration in the display. The display of time to birth and neonatal resuscitation duration intended to provide guidance on resuscitation. During the introduction of technology in Pre-MALA, there was quality improvement process established to ensure the use of technology as a result the effect of intervention was better during this phase than Pre-MALA-2. The effect of technological intervention was seen better during Pre-MALA 1 than in Pre-MALA 2 because more support and quality improvement was provided during Pre-MALA 1 to establish the technology. Furthermore, the introduction of new technology could have made the HCP more enthusiastic about ventilation during Pre-MALA 1 which then stabilized in Pre-MALA 2. Therefore, attenuation of time to first ventilation might have occurred during Pre-MALA 2.

## Methodological considerations

There are several methodological considerations and limitations to this study. Firstly, there is an attrition bias, as not all ventilated infants were included in the final analysis. To ensure that there is a nondifferential attrition bias in each phase, we compared the background and obstetric characteristic between the included and excluded population in each phase and found no significant difference. Secondly, there is a possibility of ascertainment bias during the annotation process using the Liveborn Observation app and/or ELAN video annotation tool, as different assessors may annotate the data differently. To mitigate this, the research team received training on using the Liveborn Observation app for annotation, and a standardized protocol was employed for video annotation using ELAN software. Thirdly, there is a possibility of observers inducing bias (Hawthorne effect) in resuscitation care before and after introduction of technology. Lastly, since the technology used during Pre-MALA integrates three different innovations into an infant warmer, we cannot determine which specific factor—whether the visual display of time since birth, easy access to resuscitation equipment, or video/audio recording—most influenced HCPs’ behavior in initiating ventilation more promptly. The presence of video and audio recordings during resuscitation may have impacted HCPs, encouraging them to perform more efficiently.

While simulation-based studies have demonstrated that real-time feedback can improve the time to first ventilation and reduce unnecessary suctioning,^34^ a randomized controlled study in Nepal using a real-time display in a monitor during simulated settings showed an increase in effective ventilation and a reduction in air leakage from the mask compared to simulation without a monitor.^35^ Additionally, augmented devices that display effective ventilation and air leakage during simulations have shown improvements in learning settings in Uganda, Kenya, and India.^26,36^ However, real-time feedback with the display of time since birth during ventilation has not been extensively evaluated in clinical settings in resource-limited environments, such as public referral hospitals. Although feedback after clinical performance has been shown to improve HCP performance in settings like Nepal, Tanzania, and Ethiopia as part of the Safer Birth Bundle,^23,37,38^ our study is one of the first to explore the potential impact of real-time feedback with a visual display of time since birth on reducing time to first ventilation and unnecessary suctioning. The technology used during Pre-MALA aligns with the WHO's technology-based intervention guideline for Standards-based, Machine-readable, Adaptive, Requirements-based, and Testable (SMART)^39^ approach to enhance service delivery and care measurement.

## Conclusion

The technology used during Pre-MALA study, which includes feedback on time since birth, has shown potential for reducing time to first ventilation and decreasing suctioning duration. A system with real-time feedback with video and audio recording holds promising potential for improving neonatal resuscitation performance for better outcomes in resource-limited settings. The technology is evolving and will be further developed to incorporate AI-driven real-time feedback and automated guidance for healthcare providers during resuscitation. While these preliminary results are promising, further rigorous evaluation of the technology is required to fully assess its effectiveness and potential impact on neonatal care.

## Supplemental Material

sj-docx-1-dhj-10.1177_20552076261431443 - Supplemental material for Evaluation of a novel technology for newborn resuscitation: A visual display of time since birth, video–audio recording, and ergonomic resuscitation equipment: A prospective observational design Supplemental material, sj-docx-1-dhj-10.1177_20552076261431443 for Evaluation of a novel technology for newborn resuscitation: A visual display of time since birth, video–audio recording, and ergonomic resuscitation equipment: A prospective observational design by Omkar Basnet, So Yeon Joyce Kong, Helge Myklebust, Sunil Mani Pokharel and Ashish KC in DIGITAL HEALTH

sj-xlsx-2-dhj-10.1177_20552076261431443 - Supplemental material for Evaluation of a novel technology for newborn resuscitation: A visual display of time since birth, video–audio recording, and ergonomic resuscitation equipment: A prospective observational design Supplemental material, sj-xlsx-2-dhj-10.1177_20552076261431443 for Evaluation of a novel technology for newborn resuscitation: A visual display of time since birth, video–audio recording, and ergonomic resuscitation equipment: A prospective observational design by Omkar Basnet, So Yeon Joyce Kong, Helge Myklebust, Sunil Mani Pokharel and Ashish KC in DIGITAL HEALTH

## Abbreviations

BMV: Bag-mask ventilation
ELAN: EUDICO Linguistic Annotation
EPOC: Effective Practice and Organization of Care
HCP: Healthcare provider
IQR: Interquartile range
MALA: Machine Learning Application
QI: Quality improvement
SMART: Standards-based, Machine-readable, Adaptive, Requirements-based, and Testable
SUSTAIN: Scaling Up Safer Birth Bundle Through Quality Improvement in Nepal
WHO: World Health Organization
